# Key factors for successful implementation of the National Rollover Protection Structure Rebate Program: A correlation analysis using the consolidated framework for implementation research

**DOI:** 10.5271/sjweh.3844

**Published:** 2019-07-31

**Authors:** Pamela J Tinc, Paul Jenkins, Julie A Sorensen, Lars Weinehall, Anne Gadomski, Kristina Lindvall

**Affiliations:** 1 Northeast Center for Occupational Health and Safety: Agriculture, Forestry, and Fishing, Cooperstown, NY, USA.; 2 Department of Epidemiology and Global Health, Umeå University, Umeå, Sweden.; 3 Research Institute, Bassett Medical Center, Cooperstown, NY, USA.

**Keywords:** agriculture, occupational safety, safety, scale-up, stakeholder engagement, consolidated framework for implementation research, correlation analysis, factor, implementation, National Rollover Protection Structure Rebate Program, rollover

## Abstract

**Objectives:**

On US farms, tractor overturns are the leading cause of death; however, these fatalities are preventable with the use of a rollover protection structure (ROPS). A ROPS rebate program was established in New York in 2006 to address these fatalities. Due to its success, the program expanded to six additional states before being implemented as the National ROPS Rebate Program (NRRP) in 2017. The aim of this study was to evaluate the success of the NRRP implementation using short- and long-term ROPS outcome measures and identify which components of the consolidated framework for implementation research (CFIR) correlate with these outcomes.

**Methods:**

Stakeholders involved in the NRRP implementation were surveyed at four time points, beginning at the time of the NRRP launch and then every six months. These surveys measured 14 relevant CFIR constructs. Correlations between CFIR survey items (representing constructs) and three outcome measures (intakes, funding progress, and retrofits) were used to identify CFIR survey items that are predictive of the outcomes.

**Results:**

Eight CFIR survey items were highly correlated (rho ≥0.50) with at least one of the three outcome measures. These eight CFIR survey items included four constructs: access to knowledge and information, leadership engagement, engaging (in fundraising and funding requests), and reflecting and evaluating.

**Conclusions:**

The results of this study provide important guidance for continuing the implementation of the NRRP. Similarly, these findings can inform the evaluation of other similarly structured implementation efforts and the application of CFIR in a variety of settings.

Agricultural workers face one of the highest rates of fatal occupational injuries in the US (24.0 fatal injuries per 100 000 workers, compared to the all-worker fatality rate of 3.5 fatalities per 100 000 workers) (1). Agricultural workers also face high non-fatal injury rates; the Bureau of Labor Statistics reports 5.7 non-fatal injuries per 100 workers for the agriculture, forestry, and fishing sector (2). This number, however, may be low, as it is likely injuries are considerably underreported by these occupational groups (3). Given these statistics, it is evident that agricultural health and safety is a vital component of public health efforts.

Though much has been done to identify viable solutions to common health and safety issues in agriculture, little research has focused on how to translate these solutions into widespread practice (4–6). At the time of this study, no other published efforts to scale-up agricultural safety interventions could be found. While in some cases, this is likely the result of a limited and inconsistent understanding of what it truly means to translate research into practice (5), another major barrier to such work is a lack of understanding of what works (or does not) in translating occupational health and safety innovations to practice and why translational successes or failures occur (4–6).

Despite slow progress in agricultural safety and health implementation research, implementation science efforts have rapidly expanded in the areas of clinical medicine and public health. In particular, implementation researchers have sought to develop frameworks or models that can answer key questions about the implementation process. The consolidated framework for implementation research (CFIR) is one such model that was developed in order to answer questions of what works, in what settings, and why. This study uses the CFIR to quantitatively evaluate the scaling up of an agricultural safety initiative to the national level in order to provide valuable information about implementing evidence-based agricultural safety interventions.

The CFIR was developed as a tool for use in health services, including primarily clinical medicine, but also public health (7, 8). Development of the CFIR began with a review of the literature on theories, frameworks, and models for implementation efforts, and resulted in the combination of 19 of these into one cohesive framework (8). The CFIR was selected as an evaluation tool for this study due to its comprehensive nature and its ability to be adapted for and applied to diverse settings.

The CFIR consists of five domains related to implementation efforts: individual characteristics, inner setting, outer setting, intervention characteristics, and process (7, 8). In addition, supplemental domains, including implementation outcomes and client outcomes, were developed to further describe implementation efforts (9). Each of the CFIR domains have been developed in relation to various constructs, which help describe each aspect of the implementation (table 1).

The individual characteristics domain consists of five constructs that help describe different qualities of the individuals involved in the implementation process. The inner setting is intended to describe the interactions between these individuals, as well as the immediate implementation environment. This domain uses five constructs and nine sub-constructs. The outer setting, which includes four constructs, describes the influences from stakeholders and networks outside of the inner setting implementation team.

As the name suggests, the intervention characteristics domain includes eight constructs that describe the qualities of the intervention throughout the implementation process. The process domain, which includes four constructs and four sub-constructs, logically helps to describe how the implementation occurs. Finally, implementation outcomes (five constructs) and client outcomes (two constructs) help explain whether the implementation was successful.

Because the CFIR has been developed from other implementation frameworks, the construct definitions and suggested questions have been validated individually over time (7, 10). In addition, the CFIR as a framework has been assessed for validity through a systematic review of its use (11). Despite these assessments, no applications of the CFIR to the agricultural safety setting could be found with the exception of this study’s predecessor, which served to develop the survey instrument applied in this study (12). Thus, the utility of the CFIR in agricultural safety settings has yet to be determined.

Within agriculture, tractor overturns have long been the leading cause of death (13). These fatalities can be prevented by using rollover protection structures (ROPS), which are designed to keep tractor operators in a protected zone in the event of a tractor overturn. Though ROPS became standard on newly manufactured tractors in 1985 (14), approximately 40–50% of tractors on US farms currently lack the devices (13, 15). This is in large part due to the extended lifespan of most tractors, combined with the high cost (approximately $1200 per kit) and time required to retrofit these older tractors with ROPS (16, 17). In addition, because many farmers do not feel personally vulnerable to tractor overturns, it can be difficult to justify the cost associated with retrofitting (16).

The ROPS rebate program was first launched in New York in 2006 after several years of formative research to better understand farmers’ barriers and motivators to installing the safety devices (16, 17). An initial pilot study confirmed that targeted messaging combined with technical assistance and a monetary incentive was the key to increasing the number of ROPS-equipped tractors on farms (18). Since then, more than a decade of research has been conducted and has established the ROPS rebate program as efficacious, effective, and economical (16–25).

Between 2010 and 2016, this program was expanded to, and proved effective in, six additional states: Vermont, Pennsylvania, New Hampshire, Wisconsin, Massachusetts, and Minnesota (19, 24). In all, over 2800 tractors have been retrofitted to date. Further, in annual surveys distributed to all who have retrofitted tractors through the program, farmers have self-reported 17 overturn events in which the tractor operator was likely to have been saved as a direct result of the ROPS, and more than 220 close calls and other events, such as near overturns and falling objects (26).

In 2014, researchers, industry partners, farmers, private corporations, the media, and agricultural, financial, government, and health and safety organizations from across the country joined together to form the National Tractor Safety Coalition (NTSC) and moved toward expanding the existing ROPS rebate programs (27–29). The multi-sector group, led by a steering committee of 15 representatives, has worked together over the last several years to launch the national ROPS rebate program (NRRP) in June 2017 (30).

The NRRP intervention includes three primary components: (i) a toll-free hotline and website (www.ROPSr4u.com), (ii) funding for 70% rebates toward the cost of purchasing and installing ROPS, and (iii) a social marketing campaign to encourage farmers to participate. Though a small pool of rebate funding is available at the national level, individual states are encouraged to identify state-allocated funding for rebates. Currently, national level funding is too sparse to implement the social marketing campaign in states without state-allocated funding, leaving only the tollfree hotline and website in all but the seven states previously mentioned. Once fully implemented, the NRRP will provide all of the above components, including adequate rebate funding, across all 50 states.

To achieve full implementation, a two-part strategy is currently underway to encourage adoption and sustainment of these components of the NRRP intervention. This implementation strategy includes NTSC support (information and guidance, promotion, and fundraising) and a media advocacy campaign to increase nonfarming, stakeholder support of the NRRP. Though not all 50 states have NTSC members, these implementation strategy components are available nationwide.

The aim of this study was to evaluate the success of the NRRP implementation using short- and long-term ROPS outcome measures and identify which components of the CFIR are correlated with these outcomes.

## Methods

### Data collection

#### Study timing.

The soft launch of the program, which occurred in March 2017, involved rebranding of the ROPS rebate programs into the NRRP and updating the website and program database. This occurred prior to the official launch (June 2017) so that technical issues related to the updates could be corrected before the public announcement. This study took place during a 24-month timeframe surrounding the NRRP implementation, beginning six months prior to and ending 18 months after the soft launch.

#### NRRP implementation regions.

**Figure 1.**
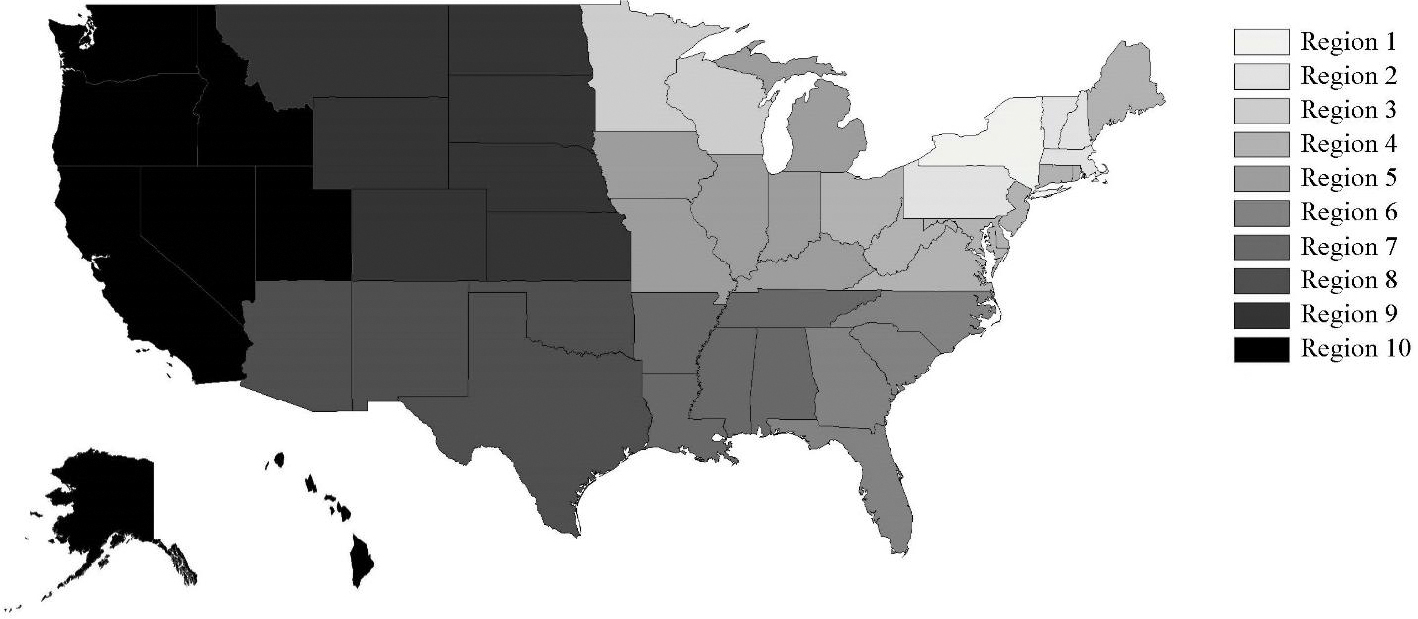
Ten national ROPS rebate program implementation regions.

The 50 states were divided into ten implementation regions (table 2 and figure 1). These ten regions were defined based on three factors: (i) geographic location, (ii) the requirement that at least one NTSC member resided in the region at each data collection point, and (iii) the availability of different NRRP intervention components.

All 50 states have access to the toll-free hotline and website. Additionally, states in regions one, two, and three had secured at least some state-allocated funding prior to March 2017 and subsequently implemented the social marketing campaign. Region one (New York) was separated from regions two and three because the program originated in New York and had been active there for the longest period of time. Regions two and three were divided based on geographic location (region two = Vermont, New Hampshire, Pennsylvania, and Massachusetts located in the Northeast, region three = Wisconsin and Minnesota located in the Midwest). As described, the remaining regions (four through ten) all have access to the toll-free hotline and website and limited national-level funding. However, state-allocated funding and social marketing campaigns are not available in these states.

#### Measurement of CFIR constructs.

In a prior study, NTSC members (N=65) were asked to score CFIR constructs from “not at all important” to “extremely important” to the NRRP implementation (12). Using these responses, program evaluators with backgrounds in clinical medicine, public health, anthropology, and implementation science developed a corresponding survey instrument containing 36 questions (termed “CFIR survey items”) that covered 14 constructs (supplementary material, www.sjweh.fi/show_abstract.php?abstract_id=3844) (12).

Surveys were conducted at four time points – March 2017, September 2017, March 2018, and September 2018 – with each survey asking participants to reflect on the six months prior. Thus, the surveys relate to four six-month periods: September 2016 through February 2017 (period one), March through August 2017 (period two), September 2017 through February 2018 (period three), and March through August 2018 (period four). The first time point, March 2017, served as a baseline measurement as it was collected at the time of the softlaunch of the NRRP. The additional three time points served to capture change over time in CFIR constructs.

Each survey was distributed to all members of the NTSC that were active in the coalition at the time of the surveys (N=56–68 at each data collection point). This number fluctuated due to factors such as NTSC members’ retiring and new NTSC members joining; however, overall the number of responses from each region remained relatively stable over time. At each data collection point, a mixed-mode survey method (31) was used to improve response rates: (i) Day 1: an invitation to participate in the survey was emailed to all participants. Included in this invitation was an explanation of the study and ethics information, as well as a link to the web-based survey. (ii) Day 8: Mailed packets were sent to non-responders. These packets included the contents of the original invitation, as well as a paper survey and an addressed, stamped envelope. (iii) Day 15: A “thank you” and reminder email was sent to the entire NTSC. (iv) Day 22: Non-responders were contacted via telephone. Participants had the option of completing the survey over the phone, if they wished. Participants were called up to three times. (v) Day 29: The survey was closed.

Participants who responded to surveys were provided with one entry per survey for a $1,000 Amazon gift card raffle. The Mary Imogene Bassett Hospital Institutional Review Board approved this study (project #2015).

### Short-term outcome measure: progress

For each six-month survey period, the project team (consisting of two researchers, the hotline coordinator, and the marketing coordinator) assigned each state a progress score between 0–6, where 0=no program and no known fundraising activity and 6=current program with sufficient funding (table 3). program records (such as email correspondence and meeting notes) were used to determine these scores. This seven-level outcome was termed “progress.”

### Long-term outcome measures: Intakes and retrofits

Using the NRRP hotline, the number of individuals who sign up for the program (“intakes”) as well as the number of tractors that were retrofitted (“retrofits”) are regularly tracked. These data were obtained for each of the ten intervention regions between 1 September 2016 and 31 August 2018. For individuals completing both an intake and a retrofit during the study period, only the retrofit was counted; however, the date used was reflective of the intake date. For example, if a participant completed an intake in January 2017 and then completed the retrofit in March 2017 they were counted as a retrofit in January 2017 (period 1).

### Data analysis

The change in each of the 36 CFIR survey items was compared between the ten regions over the four time periods using a four by ten mixed analysis of variance (ANOVA). The F-test for the interaction of region by time was used to evaluate whether the change in each item over time was significantly different between the regions. This same four by 10 model was used to analyze the changes in the three outcomes: retrofits, intakes, and progress scores.

The ANOVA models described above did not find significant time or region by time interaction effects. Because of this, the data were aggregated across the four time periods for each region by taking the mean value. This was done both for the CFIR survey items and the three outcomes.

The correlations between these time-averaged CFIR survey items and the three outcomes were analyzed using Spearman’s rho. Due to the small sample size (N=10 regions), these correlational analyses had limited statistical power, which presented the possibility that CFIR survey items with correlations of practical relevance were not necessarily statistically significant. Therefore, any Spearman’s correlation between a CFIR survey item and an outcome of ≥0.50 was deemed to be predictive of the outcome regardless of the significance level.

## Results

### Descriptive statistics

The response rates for the surveys were 50.0%, 49.2%, 60.7%, and 67.9% for the first, second, third, and fourth surveys, respectively. The mean scores for each CFIR item can be found in table 4.

Over the study period, there was a total of 1445 intakes and 404 retrofits across all regions. Average progress, intakes, and retrofits are shown for each region and period in table 5. As can be seen in the table, most of the intakes, nearly all of the retrofits, and the highest progress scores are in the regions 1, 2, and 3, all of which had obtained funding for rebates prior to this study.

### Correlation analysis

Table 6 shows the correlations between CFIR survey items and outcomes of ≥0.50. For clarity, these CFIR survey items are numbered in the table and will be referred to by number in the text.

As shown in table 6, a total of eight CFIR survey items were correlated with one or more of the three outcome measures. CFIR survey items two and three were correlated with both progress and retrofits, and CFIR survey items one, four, six, and seven were correlated with all three outcome variables. These eight CFIR survey items covered four constructs in two domains: access to knowledge and information (inner setting), leadership engagement (inner setting), engaging (process), and reflecting and evaluating (process). Those that correlated with all three outcome variables included CFIR survey items related to access to knowledge and information (inner setting) and engaging (process).

## Discussion

CFIR survey items that are highly correlated with all three outcomes may be the most important in moving forward with the NRRP implementation. These CFIR survey items include NTSC members’ belief that part of their role is to raise funds for the program (CFIR survey item six) as well as their active role in submitting funding applications (CFIR survey item seven). This is intuitive, in that the short-term outcomes and the success of the program reflect fundraising for rebate dollars. As such, those states with pre-established funding (regions 1, 2, and 3) were, as expected, the most successful in terms of outcomes. Despite this, some small successes occurred in other regions, including movement toward higher progress scores and a handful of intakes in each region for each time point. Progress scores >0 indicate that the efforts put forth to implement the NRRP have been marginally successful. However, it is also important to note that securing funding (ie, reaching a progress score of 4 or 6) for this type of initiative can take longer than the time allotted in this study. Thus, if reassessed after another year, it is possible that progress scores would be higher.

Once state-allocated rebate funding has been secured, the social marketing campaign can be implemented in that state, thus triggering additional farmers to complete intakes and subsequently retrofit their tractors. Though there are intakes reported in each study region, the numbers are much lower in regions 4 through 10. Still, these marginal successes indicate farmer interest in the NRRP, as without direct advertising in unfunded regions, farmers could only have discovered the program as a result of their own attempts to retrofit tractors.

In addition to CFIR survey items related to fundraising, the item “program materials are engaging” (item one) was highly correlated with all outcomes. This finding suggests that such materials (including both those that are targeted at stakeholders who can help secure state-allocated funding as well as farmers who may participate) may motivate stakeholders to take action by fundraising, signing up for the NRRP themselves, or further disseminating NRRP materials to others, and make those actions easier. This is supported by the basic premise of the construct (32), as well as principles of social marketing (33, 34), which suggest that materials that are easier to engage with are more likely to promote the anticipated behavior. This principle was demonstrated in developing the social marketing messages to increase farmer participation in the ROPS rebate programs (22, 25).

Finally, the CFIR survey item “stakeholders understand their role to include providing feedback about implementation activities and fundraising,” (item four) was also highly correlated with all three outcome measures. In these cases, individuals who are providing feedback about program implementation and fundraising may feel a greater sense of ownership, thus motivating them to be further involved. Ownership has often been cited as a key motivator for employees, stakeholders, and other populations (35–38), as it facilitates the transformation of individuals’ roles and responsibilities within an organization or organized effort (38). In the public health sector, community coalitions have focused on ownership as an indicator of increased engagement and improved health outcomes (39).

Two CFIR survey items were highly correlated with at least two outcome measures, again, indicating practical significance. Two measures of leadership engagement, “the NTSC steering committee encourages members to be involved in implementing the program,” and “the NTSC steering committee is supportive of the program,” (items two and three) were both highly negatively correlated with progress and retrofits. This is perhaps the most surprising finding, in that one would expect the opposite to occur – a more highly engaged leadership should be correlated with improved outcomes. In a qualitative portion of this study (Tinc et al, forthcoming); the study team explored this finding in interviews with steering committee, general NTSC, and non-NTSC stakeholders who identified two possible explanations. First, it was suggested that because the steering committee is comprised of well-known and well-respected individuals in the agricultural research community, the program may be seen as a “top-down” approach. Thus, others may be less motivated to engage in the implementation process by raising funds or promoting the program in their state. This is consistent with the stakeholder engagement literature, which recommends collaborative partnerships, reciprocal relationships, and co-learning, among other strategies for increasing engagement (40, 41). However; some psychology research contradicts this observation by suggesting that individuals are more likely to engage in behaviors (such as being involved in the NRRP implementation) that are promoted by authority figures, which could include members of the steering committee (42). Second, it was suggested that the states with active and sufficiently funded programs may have gotten to that point without the help of steering committee members. Thus, those who have state-allocated funding in place may not place value on steering committee members’ contributions.

The CFIR survey items “NTSC updates are helpful in allowing me to reflect upon progress toward implementation of the program” and “participation in implementation events (perceived role),” (items eight and five) were both highly correlated with intakes, but not with the other two outcome variables. This might suggest that both of these activities are enough to encourage stakeholders to spread the word about the program to the target population, but do not motivate stakeholders to dedicate time to fundraising activities.

The results of this study are immediately important, in that they provide guidance for continuing the implementation of the NRRP. Based on these results, it is important to consider barriers to stakeholder engagement and how to remove those barriers, especially for NTSC members and stakeholders in regions with low numbers of intakes and retrofits. In addition, the alarmingly high negative correlations between CFIR survey items related to steering committee engagement and outcome variables suggest that more needs to be done to encourage full-NTSC communication and excitement about the NRRP. A social networking analysis focused on NTSC member interactions is currently underway and will provide valuable information related to NTSC member communications and collaboration.

In addition to the primary findings of this study, it is also relevant to comment on the utility of the CFIR in occupational safety settings, such as this one. A prior study by the research team demonstrated that while the CFIR was applicable in agricultural safety settings, there were some challenges applying the CFIR to scale-up initiatives versus single site implementation studies (12). This sentiment was echoed in a review of CFIR use published in 2016 (11). The results of this study indicated that initial assessments of the utility of the CFIR within agricultural safety settings were accurate. However, further assessment of the CFIR’s utility using other occupational safety interventions would be important in confirming this finding.

### Limitations

There are four main limitations to this study. First, only constructs described by the CFIR were evaluated for correlation with ROPS outcomes and only a portion of the total possible CFIR constructs were included in this portion of the study. Thus, there may be additional factors relevant to the implementation of the NRRP that were not explored in this study. However, additional constructs would have made the survey longer and impacted response rates so the decision was based on achieving a balance between these potential research design challenges. Additionally, qualitative interviews with NTSC members and non-members have been used to elicit additional information about the program implementation and clarify survey responses (Tinc et al, forthcoming).

Second, the sample size for this study is quite small, which is primarily due to the size of the NTSC and location of its members. NTSC members do not exist in all states, and thus survey responses were also not available for all states and so regions were used as the units of analysis. It is possible that if study results were to be compared between individuals or states, the analyses presented here might result in different outcomes. Thus, these conclusions should be considered in light of this fact.

Third, the average response rate for the CFIR surveys was only 57%. While this is a relatively promising response rate on such surveys, it is possible that the results presented here could differ from the results had all NTSC members responded to the various survey requests. In particular, the individuals who did not respond to the survey requests may have drastically different viewpoints of the implementation process. It is also possible that, had all NTSC members responded to all survey requests, the correlations presented here may have been different.

Finally, it should be acknowledged that the study, being observational in nature, cannot establish cause and effect. Whether or not a change in a CFIR item will produce a change in outcomes such as intakes and retrofits, cannot be established with certainty.

## Concluding remarks

This study provided insight into key factors that have influenced the NRRP implementation, including both those that have hindered and helped the effort. In continuing with the NRRP implementation, it may be helpful to consult the stakeholder and employee engagement literature for guidance and strategies. For example, modifying communication strategies, engaging in shared leadership strategies, and simultaneously targeting emotion, beliefs, and behaviors could help improve stakeholder engagement (37, 38). Further work to evaluate changes in the CFIR survey items discussed in this manuscript would be beneficial to assess the impact of such strategies.

Outside of the NRRP implementation, these results could be useful for others hoping to attempt similarly structured implementation initiatives. First, the evaluation structure utilized in this study could be applied in other areas of occupational and public health. Second, the key findings may be useful to consider when planning implementation studies that will require involving a wide variety of stakeholders.

## Supplementary Material

Appendix

## Figures and Tables

**Table 1.** The consolidated framework for implementation research (CFIR) and supplemental outcome domains and constructs adapted from Damschroder et al (8) and Proctor et al (9).

Domains	Constructs

CFIR	
Individual	Knowledge and beliefs about the intervention
characteristics	Individual stage of change
	Individual identification with the organization
	Self-efficacy
	Other personal attributes
Inner setting	Culture
	Implementation climate
	Networks and communication
	Readiness for implementation
	Structural characteristics
Outer setting	Cosmopolitanism
	External policy and incentives
	Patient needs and resources
	Peer pressure
Intervention	Adaptability
characteristics	Complexity
	Cost
	Design quality and packaging
	Evidence strength and quality
	Intervention source
	Relative advantage
	Trialability
Process	Engaging
	Executing
	Planning
	Reflecting and evaluating
Outcomes	
Implementation	Acceptability
	Adoption
	Appropriateness
	Feasibility
	Penetration
Client	Sustainability
	Satisfaction

**Table 2.** Ten national ROPS rebate programs implementation regions and the intervention components currently available in each region.

Region	States	Hotline & website	Rebate funding	Social marketing

1	New York	X	X	X
2	Vermont, New Hampshire, Pennsylvania, Massachusetts	X	X	X
3	Wisconsin, Minnesota	X	X	X
4	Maine, Rhode Island, Connecticut, Delaware, Maryland, New Jersey, Virginia, West Virginia, Ohio	X		
5	Michigan, Illinois, Indiana, Iowa, Missouri, Kentucky	X		
6	North Carolina, South Carolina, Georgia, Florida	X		
7	Tennessee, Mississippi, Alabama, Arkansas, Louisiana	X		
8	Texas Oklahoma, New Mexico, Arizona	X		
9	Nebraska, Colorado, Wyoming, Kansas, South Dakota, North Dakota, Montana	X		
10	Washington, Oregon, California, Idaho, Nevada, Alaska, Hawaii	X		

**Table 3.** Rubric for scoring progress in securing funding for the National ROPS Rebate Program.

Score	Description

0	No program and no known fundraising activity
1	No program but information requested
2	No program but planning for fundraising
3	No program but actively pursuing funding
4	Current program with insufficient funding
5	Current fundraising to supplement funding
6	Current program with sufficient funding

**Table 4.** Mean consolidated framework for implementation research (CFIR) survey item scores for each region across all four periods.

Domain	Construct	CFIR survey item	Region									
			1	2	3	4	5	6	7	8	9	10

Individual characteristics	Knowledge and beliefs about the intervention ^a^	It is feasible to implement the program.	4.5	4.2	3.9	4.7	3.7	4.0	4.6	4.0	4.4	4.0
		The implementation of the program is going well.	4.1	4.0	3.6	4.0	3.3	3.3	4.0	3.8	3.8	3.8
Inner setting	Access to knowledge and information ^a^	i Program information and materials are appropriate.	4.8	4.7	4.2	4.8	4.4	4.0	4.4	5.0	4.4	4.4
		Program information and materials are engaging.	4.8	4.4	4.3	4.5	4.3	3.0	4.4	4.0	4.3	4.1
	Available resources ^a^	I have the resources I need to promote the program in my role.	4.7	3.7	3.4	3.7	3.2	2.8	4.0	4.0	3.6	3.1
	Leadership engagement ^a^	The Coalition Steering Committee is supportive of the program.	3.9	4.3	4.1	4.5	4.6	5.0	4.8	4.5	4.3	4.1
		The Coalition Steering Committee encourages coalition members to be involved in implementing the program.	3.6	4.2	4.1	4.5	4.4	5.0	4.5	4.8	4.3	4.1
	Tension for change ^a^	It is important that the program is implemented now.	5.0	4.6	4.4	4.9	4.3	3.8	4.5	5.0	4.3	4.1
Outer setting	Cosmopolitanism ^a^	My employer encourages me to network with colleagues outside of my own setting.	4.4	4.1	4.1	4.0	3.7	4.8	4.8	4.0	4.2	3.6
	Farmer needs and resources ^a^	Once implemented, the program will meet the needs of my organization’s target population.	4.7	3.9	3.8	4.2	3.5	4.5	4.4	3.3	3.7	3.9
Intervention Characteristics	Cost ^a^	The cost of the program has not prevented it from being implemented in my state.	4.7	3.4	2.9	2.8	2.7	1.8	3.1	3.0	3.0	3.6
	Design quality and packaging ^a^	Program materials (including the website, promotional materials, and information packets) are of high quality.	4.8	4.8	4.2	4.7	4.4	4.3	4.4	5.0	4.4	4.4
	Evidence strength and quality ^a^	Influential stakeholders (such as funders, manufac-turers, or other influential individuals) are supportive of the program.	3.8	4.2	3.9	3.8	3.3	3.3	4.3	3.5	3.6	3.7
Process	Reflecting and evaluating ^a^	Coalition updates are helpful in allowing me to reflect upon progress toward implementation of the program.	4.4	4.3	4.4	4.4	4.3	4.3	4.6	4.0	4.1	4.3
	Engaging (perceived role) ^b, c, d^	Monitoring progress so that I can stay informed.	0.8	0.9	1.0	0.8	0.9	0.8	1.0	1.0	0.9	0.6
		Providing feedback about activities that others are planning and carrying out.	0.8	0.5	0.8	0.6	0.6	0.3	0.7	0.8	0.5	0.4
		Sharing promotions and materials with partners outside of the coalition.	1.0	0.8	0.9	0.8	0.6	0.5	1.0	0.8	0.8	0.6
		Helping plan implementation activities such as events and fundraising.	0.7	0.2	0.4	0.5	0.1	0.3	0.5	0.7	0.1	0.1
		Participation in implementation events.	0.7	0.4	0.4	0.3	0.2	0.3	0.7	0.0	0.2	0.3
		Participation in fundraising.	0.6	0.3	0.3	0.2	0.1	0.0	0.2	0.0	0.2	0.1
	Engaging (actual activity) ^c, d, e^	Read coalition updates, information, or materials.	2.8	1.6	2.0	1.5	1.5	1.5	2.3	1.0	1.6	1.7
		Attended a coalition webinar or conference call.	1.4	0.9	0.8	0.5	0.8	0.8	1.4	0.8	0.9	1.2
		Attended a coalition in-person meeting.	0.7	0.1	0.3	0.0	0.0	0.0	0.4	0.0	0.1	0.3
		Attended an event on behalf of the coalition or program.	0.8	0.0	0.4	0.0	0.2	0.0	0.6	0.3	0.3	0.4
		Provided feedback or suggestions on Coalition activities or materials via email or a one-on-one phone call.	2.0	0.8	0.7	0.6	0.7	1.0	1.4	1.0	0.6	0.9
		Provided feedback or suggestions on coalition activities or materials during a coalition webinar, conference call, or in-person meeting.	1.9	0.7	0.6	0.3	0.7	1.0	1.3	0.8	0.3	0.7
		Shared program information or promotions with a group of individuals via social media, email distribution lists, or newsletters.	1.5	0.7	1.2	0.5	0.7	0.8	1.5	0.3	0.9	1.1
		Incorporated program information into a presentation or report that you were putting together for another purpose.	0.9	0.8	1.0	0.8	0.5	0.3	1.1	0.5	0.3	1.2
		Had a conversation about the program with an individual(s) not involved in the Coalition.	2.4	1.2	1.2	1.0	0.8	0.5	1.5	0.8	0.9	1.9
		Served as a spokesperson specifically for the Coalition or program (through interviews, presentations, etc.).	1.2	0.2	0.4	0.1	0.0	0.5	0.8	0.0	0.1	0.0
		Recruited new members to the coalition or connected coalition members with new partners.	0.5	0.0	0.5	0.0	0.1	0.0	0.6	0.0	0.2	0.3
		Helped arrange or plan coalition activities or events.	1.3	0.0	0.2	0.0	0.0	0.0	0.4	0.0	0.0	0.1
		Helped submit a funding or resource request for the program.	0.7	0.2	0.2	0.0	0.1	0.0	0.1	0.0	0.1	0.1
		Met with potential funders to discuss funding the program.	0.7	0.3	0.1	0.3	0.0	0.0	0.1	0.5	0.2	0.4
Implementation outcomes	Acceptability ^a^	The program is an acceptable response to tractor overturn fatalities.	4.9	4.5	4.4	5.0	4.2	5.0	4.9	4.8	4.6	4.0
Client outcomes	Sustainability ^a^	The program is sustainable.	4.3	3.6	2.9	3.5	3.0	2.5	4.0	3.5	3.5	3.4

a Measured on a five-point Likert scale from 1=strongly disagree to 5=strongly agree.

b Measured as a binary variable, where 0=not part of the stakeholders’ role and 1=the activity is part of the stakeholders’ role.

c All measures relate to the construct engaging; however, in the survey, participants were asked to share what their perceived role in implementation was compared to the activities that they are actually engaged in. For clarity, this distinction was also made in this table.

d While all of the CFIR survey listed here refer to the “engaging” construct, there are different levels and types of engagement. Thus, for each perceived and actual roles, the various activities listed are ordered based on the level of engagement. These orders were determined in discussions with the Program team.

e Measured on a six-point Likert scale from 0=did not do at all to 5=participate in more than 1–2 times per week.

**Table 5.** Outcomes (average progress scores, number of intakes, and number of completed retrofits) by region number (as defined in table 2) and period, where period 1 =September 2016-February 2017, period 2 = March-August 2017, period 3 = September 2017-February 2018, and period 4=March-August 2018.

Region	Progress				Intakes^a^				Retrofits ^a^			
	
	Period 1	Period 2	Period 3	Period 4	Period 1	Period 2	Period 3	Period 4	Period 1	Period 2	Period 3	Period 4

1	6.00	6.00	6.00	6.00	98	68	97	75	25	38	53	20
2	4.25	4.25	4.75	4.75	6.5	10	8.75	8.75	0.5	0.75	1	0.75
3	6.00	6.00	6.00	6.00	92	70.5	63	72	44.5	36	25	11.5
4	0.11	0.22	0.67	0.78	0.3	2.9	3.0	1.6	0	0	0	0
5	0.67	0.33	0.33	0.33	3.2	3.7	6.3	3.8	0	0	0.2	0
6	0.00	0.00	0.00	0.25	1.75	1.5	2.25	2.5	0	0	0	0
7	0.20	0.20	0.00	0.00	1.2	1.4	1.0	4.8	0	0	0	0
8	1.00	0.00	0.00	0.00	0.25	2.0	1.75	2.0	0	0	0	0
9	0.33	0.00	0.00	0.33	0.5	1.33	2.33	1.0	0	0	0	0
10	0.43	0.43	0.43	0.29	0.14	0.28	1.0	1.71	0	0	0	0

a Intakes and retrofits are shown as an average per period per state in the region.

**Table 6.** Correlation between consolidated framework for implementation research (CFIR) survey items and outcomes: coefficients and P-values for all correlations where rho≥0.50. Significant P-values (≤0.05) are bolded.

Domain	Construct	CFIR survey item		Progress		Intakes		Retrofits	
	
		#	Item Description	rho	P-value	rho	P-value	rho	P-value

Inner setting	Access to knowledge and Information	1	The program materials are engaging	0.687	0.028	0.648	0.043	0.567	0.088
	Leadership engagement	2	The Coalition Steering Committee encourages members to be involved in implementing the program	−0.729	0.017			−0.731	0.016
		3	The Coalition Seering Committee is supportive of the program	−0.705	0.023			−0.574	0.083
Process	Engaging (perceived role) ^a^	4	Providing feedback about activities that others are planning and carrying out	0.571	0.084	0.564	0.090	0.594	0.070
		5	Participation in implementation events			0.500	0.141		
		6	Participation in fundraising	0.691	0.027	0.543	0.105	0.687	0.028
	Engaging (actual role) ^a^	7	Submitted a funding or resource request for the program	0.586	0.075	0.505	0.137	0.756	0.012
	Reflecting and evaluating	8	Coalition updates are helpful in allowing me to reflect upon progress toward implementation of the program			0.583	0.077		

a All measures relate to the construct “engaging”; however, in the survey, participants were asked to share what their perceived role in implementation was compared to the activities that they are actually engaged in. For clarity, this distinction was also made in this table.
