# Supplementary material for: Key factors for successful implementation of the National Rollover Protection Structure Rebate Program: A correlation analysis using the consolidated framework for implementation research
Source: Scand J Work Environ Health. Author manuscript; Available in PMC 2026 Apr 13. (PMC13075194; doi:10.5271/sjweh.3844)
Supplement: Appendix [file NIHMS2155944-supplement-Appendix.pdf]

Key factors for successful implementation of the National Rollover Protection Structure Rebate Program: A correlation analysis using the consolidated framework for implementation research [1]

by Pamela J Tinc, MPH,[2] Paul Jenkins, PhD, Julie A Sorensen, PhD, Lars Weinehall, MD, Anne Gadomski, MD, Kristina Lindvall, PhD

1. *Appendix: survey instrument*
2. *Correspondence to: Pamela J Tinc, Northeast Center for Occupational Health and Safety: Agriculture, Forestry, and Fishing, 1 Atwell Road, Cooperstown, NY 13326, USA. [E-mail: pam.tinc@bassett.org]*

Implementing the national ROPS rebate program period

The purpose of this survey is to help evaluate how the implementation of the National ROPS Rebate Program (NRRP) is going. Please base your answers on your own opinions (not what you believe other NTSC members or your organization think).
Important Acronyms and Definitions:

1) NRRP: National ROPS Rebate Program

2) NTSC: National Tractor Safety Coalition (NTSC members include researchers, engineers, insurance companies, government organizations, agricultural organizations, health and safety organizations, and farmers/farm advocates. The Steering Committee, which oversees the Coalition, includes representatives from many of these sectors.)

Thank you for participating in this survey! If you have any questions, please feel free to contact

Pam Tinc at pam.tinc@bassett.org or 800-343-7527, ext. 2230.

NOTE: In order to earn a raffle entry, you must complete the entire survey by midnight on date. As a reminder, this is the seventh in a series of eight surveys. For each one that you complete, you will receive one raffle entry for a $1,000 Amazon gift card.

1. Please indicate your level of agreement with the following statements:

	Strongly Disagree	Somewhat Disagree	Neither Agree nor Disagree	Somewhat Agree	Strongly Agree
**The NRRP is an acceptable response to tractor overturn fatalities.**					
**It is feasible to implement the NRRP.**					
**It is important that the NRRP is implemented now.**					
**The implementation of the NRRP is going well.**					
**The cost of the NRRP has not prevented it from being implemented in my state.**					
**The NRRP is sustainable.**					
**Once implemented, the NRRP will meet the needs of my organization's target population.**					
**I have the resources I need to promote the NRRP in my role.**					
**NRRP materials (including the website, promotional materials, and information packets) are of high quality.**					
**NRRP information and materials are appropriate.**					
**NRRP information and materials are engaging.**					
**NTSC updates are helpful in allowing me to reflect upon progress toward implementation of the NRRP.**					
**The NTSC Steering Committee is supportive of the NRRP.**					
**The NTSC Steering Committee encourages members to be involved in implementing the NRRP.**					
**Influential stakeholders (such as funders, manufacturers, or other influential individuals) are supportive of the NRRP.**					
**My employer encourages me to network with colleagues outside of my own setting**					

In the space below, please include any further explanation of your responses that you feel we should know, especially for points that you disagreed with.

2. What do you feel your role is in implementing the National ROPS Rebate Program? (Please check all that apply)

- □ Monitoring progress so that I can stay informed
- □ Providing feedback about activities that others are planning and carrying out
- □ Sharing promotions and materials with partners outside of the Coalition
- □ Helping plan implementation activities such as events and fundraising
- □ Participation in implementation events
- □ Participation in fundraising
- □ Other (please specify):

3. In the past six months, how often have you done each of the following?

	Not at all	1-2 times	1-2 times per month	1-2 times per week	More than 1-2 times per week
**Read NTSC updates, information, or materials.**					
**Attended a NTSC webinar or conference call.**					
**Attended a NTSC in-person meeting.**					
**Attended an event on behalf of the NTSC or NRRP.**					
**Provided feedback or suggestions on NTSC activities or materials via email or one-on-one phone call.**					
**Provided feedback or suggestions on NTSC activities or materials during a NTSC webinar, conference call, or in-person meeting.**					
**Shared NRRP information or promotions with a group of individuals via social media, email distribution lists, or newsletters.**					
**Incorporated NRRP information into a presentation or report that you were putting together for another purpose.**					

**Had a conversation about the NRRP with another individual(s).**	
**Served as a spokesperson specifically for the NTSC or NRRP (through interviews, presentations, etc.).**	
**Recruited new members to the NTSC or connected NTSC members with new partners.**	
**Helped arrange or plan NTSC activities or events**	
**Submitted a funding or resource request for the NRRP.**	
**Met with potential funders to discuss funding the NRRP.**	
**Other (Please specify):**	

Thank you for your response!
